# Report of a Case of Dislocation upon the Dorsum Ilii, Reduced after Twenty-Five Days, by Manipulation

**Published:** 1865-01

**Authors:** A. M. Fauntleroy

**Affiliations:** Surgeon-in-Charge, General Hospital, Staunton, Va.


ART. III.—*Report of a Case of Dislocation upon the Dorsum Ilii, reduced after Twenty-five Days, by Manipulation.* By A. M. FAUNTLEROY, Surgeon-in-Charge, General Hospital, Staunton, Va.

Ordnance Sergeant Nunn, æt. 25, admitted October 12th, 1864, with dislocation of the left femur upon the dorsum ilii:

*History.*—The accident occurred at Mount Jackson, September 22, 1864. This soldier was riding in an ordnance wagon, which was upset by some accident, and he was thrown upon his face; a portion of the ammunition fell upon his hip and thigh.

*Signs of Dislocation.*—Loss of rotundity of left buttock; left femur was slightly flexed, and rotated with adduction; the knee resting upon the patella of the right thigh and the ball of the big toe upon the dorsum of right foot. Great pain was experienced upon an attempt to extend the thigh, and considerable manual traction made without chloroform, failed to dislodge the head of the bone from its abnormal position. Manipulation was decided upon, and with the aid of chloroform, was successfully employed. The patient was placed upon the table, upon his right side, the leg of the injured side being flexed upon the thigh, and the latter flexed strongly upon the body, with slight abduction. The adhesions which the head of the bone had contracted were broken up at the first attempt, and the bone assumed a new position in the great ischiatic notch, somewhat sustaining the views of Boyer, that this latter dislocation is always consecutive in occurrence.

The head of the bone was felt in its new position, shortening slightly lessened, and foot not quite so much inverted. A second essay was made, the manipulation differing from that used in the first instance by the traction made upon the femur at the moment of its flexion upon the body, and its abduction, by which means the head of the bone was lifted over the edge of the acetabulum, and returned into the cotyloid cavity without snap, and the limb was extended and found to be normal in position and length.

He was retained in bed for a few days, and ordered to apply liniment ammoniæ over the gluteii muscles. The soldier was furloughed on the 27th of October for thirty days, still complaining of considerable soreness of the hip. This reduction was accomplished before the medical staff of the hospital. I was assisted by Surgeons R. F. Baldwin, S. B. Fisher, and Assistant Surgeons Bush and Westmoreland.

To notice in this connection some interesting points in regard to coxo-femoral dislocation, and give a cursory view of the literature of manipulation, will not, I hope, be deemed inappropriate by the readers of the "Journal."

It is a familiar fact with the profession that there are four principal dislocations of the coxo-femoral articulation. In 104 cases they occur in the following ratio of frequency: 55 upon dorsum ilii; 22 into the great ischiatic notch; 13 upon the foramen thyroideum; 8 upon the pubes. Chelius and and Samuel Cooper reverse the order of the two last varieties.

Sir Astley Cooper, whose work upon luxations is most favorably known to the profession, and whose experience is of admitted value, estimates that out of 20 cases, 12 would be upon the dorsum ilii; 5 in the ischiatic notch; 1 upon the pubic bone. Gross attributes the great frequency of the dislocation upon the dorsum ilii rather to the position in which the thigh is placed at the time of accident, than to any differences of structure in the hip ligaments at particular portions of their extent.

This explanation but partially satisfies the facts in the premises. To sustain this assertion we must beg tolerance while presenting some especial features in the anatomy of the capsular ligament, familiar with all in the profession. It is thicker, anteriorly and superiorly, than elsewhere, for it is strengthened by a bundle of fibres which descend from the anterior inferior spinous process of the ilium, and by some fibres from that portion of the rectus femoris, which is attached to the superior part of the edge of acetabulum. Hence we are not surprised to find the femur but seldom dislocated in this direction. The capsular ligament is feeblest at its external and inferior aspect, and the muscles which rotate and abduct the thigh are but slightly restrained in their action by the muscles on the inner part of the femur. These anatomical features of the hip-joint, viewed in connection with the fact that the position most favorable for dislocation upon the dorsum ilii is instinctively assumed, in enlarging the polygon of gravity, in the attempt to avert a fall or lessen its violence, which is sometimes accomplished at the expense of dislocation.

*Causes*—Violent forcing the thigh in extreme abduction or adduction, with rotation inwards, especially, when at the same moment, the femur is driven upwards and backwards.

*Pathological Anatomy.*—In every instance the capsule is lacerated—posterior half especially; the round ligament is ruptured, and some of the smaller muscles either stretched or torn. Glutaeus maximus medius and minimus are pushed up and folded, the head of the bone resting within the fibres of the deep muscles. Triceps adductor stretched (Hamilton). All the muscles upon the outer portion of the thigh are relaxed, excepting the pyriformis, gemelli, obturatores and quadratus, the limb is consequently shortened, and one would expect at first blush to find the femur rotated outwards, since the muscles we have mentioned are outward rotators; but this is not the case, for the knee and the point of the foot are always turned inwards, which appears to depend upon the influence exercised by that portion of the capsular ligament which comes from the anterior inferior spinous process of the ilium. Fenner thus reports a case of autopsy in regard to one who died after dislocation upon the dorsum ilii: "Extensive ecchymosis existed; on raising the glutaeus maximus and medius, the naked head of the femur was found upon the dorsum ilii—ligamentum teres hanging to it, but partially torn off. Portions of the obturator externus, pyriformis and gemelli were ruptured. The capsule was torn through to one-half of its extent. The external muscles being cut away, the thigh could not be brought down. The iliacus internus and psoas magnus were then severed, which permitted it to descend a little, but the head could not be replaced. The triceps adductor was divided without effect. The ilio femoral ligament was found tensely stretched. All the muscles between the pelvis and the thigh were severed, and still it was impossible to reduce the dislocation. The head of the femur could not be forced back through the rent in the capsule, and it was not until the opening had been enlarged that the reduction was completed." Fenner, of New Orleans, and Moore, of Rochester, and others, attribute the difficulty of the reduction to the obstacle offered by the capsule, the opening in which ligament being too small to admit of reduction; and it is advised to move the limb in different directions, in order to increase the cleft in the ligament. The muscular system being held in abeyance by chloroform, and the reduction readily accomplished, the inference is a very fair one, that the capsule was so much torn as to offer no obstacle to reduction. This conclusion, to which we are led by inductive reasoning, is sanctioned and confirmed by the evidence gained from repeated autopsies. When, after the use of chloroform and nauseants, reduction is impracticable, the inference, from the same course of reasoning, is that the capsule is so slightly torn that the head of the bone cannot be made to return through the small rent or button-hole thus made.

*Differential Diagnosis.*—From intra capsular fracture the posterior dislocations are readily recognized, as in the former the limb is shortened, rotated outwards, and the foot everted. Intra capsular fracture could scarcely be counfounded with either of the anterior dislocations; the position of the head of bone in latter event, the general lengthening of the limb will readily lead to a correct diagnosis.

From morbus coxarius can be readily distinguished (though mistakes have been made) by the age of the patient and the commemorative signs.

*Age of Greatest Frequency.*—The *Gazette Medicale* mentions a case of dislocation upon the dorsum ilii in a child, eighteen months old. Kirby reports a case in a child three years old. Mr. Image reported to the Suffolk Branch of the Provincial Medical and Surgical Association a case of dislocation upon the dorsum ilii in a boy three and a half years old. Mr. Image reported the case "in consequence of a charge having been made against a neighboring surgeon, of pretending to reduce a dislocation of the femur on the dorsum ilii of a child four years old. It was agreed and proved by the authorities that no such case *was recorded*, and therefore had not occurred, and that seven years was the earliest period at which this accident had taken place." J. M. Sitten, of Texas, reports a case in a girl four years old, reduced by manipulation, (New York Medical Journal, March, 1852); Norris reports a case at eleven years, (American Journal Medical Sciences, February, 1839); Gibson reports a case at twelve years, (Gibson's Surgery, Vol. I, p. 389.) Hamilton's Analysis, under 13 years, 15 cases; between 13 and 30 years, 32 cases; between 30 and 45 years, 29 cases; between 45 and 60 years, 7 cases, the youngest being two years and one month; the average fraction less than thirty-four years. This dislocation is most frequent between the ages of 25 and 45 years. It is not likely to occur in advanced life, as the brittleness of the bones makes fractures more possible. In early life it is unusual, because the violence sufficient to produce dislocation is more apt to occasion separation of epiphyses of bone.

*Period of Reduction.*—Mr. Liston reduced, without assistance, a dislocation upon the dorsum ilii, two or three minutes after it occurred. This is probably an unexampled case, but proves that the earlier the reduction is attempted the less resistance is offered by the muscles. Fergusson has not witnessed a successful effort after three weeks.

Sir Astley Cooper has described instances after four or five weeks. Breschet mentions reduction after seventy-eight days; and in the Memoires de l'Academie Royale de Chirurgie de Paris, Vol. V, p. 529, is related a dislocation of the hip, reduced after two years. Authors generally agree that reduction may be attempted within three months, but that the dangers and difficulties of reduction, after such a lapse of time, should be plainly stated to patient.

*Treatment by Manipulation and Extension.*—Hippocrates says: "In some the thigh is reduced with no preparation, with slight extension directed by the hands, and with slight movement; and in some the reduction is effected by bending the limb at the time of making rotation." Richard Wiseman wrote of reduction by manipulation in 1676, (Hamilton); Richard Boulton called attention to this subject in 1713, (Boulton's Practical Surgery).

Thomas Anderson, of Scotland, reported a case thus treated by himself in 1772, (Anderson's Medical Commentaries); Daniel Turner reports a case in 1742, (Turner's Art of Surgery); Pontean draws attention to this subject in 1783, (Vidal); Dr. Philip Syng Physick, of Philadelphia, reduced a case by manipulation in January, 1811, before his class; Dr. Nathan Smith, Professor of Surgery in New Haven Medical College, taught, in 1815, that this was the only proper way of reducing this dislocation, (Dorsey's Surgery); Dr. Nathan R. Smith, (his son), Professor of Surgery in Baltimore Medical College, has always taught similarly. Similar directions are given by Palleta in 1818. Dr. Tuke Home, of Boston, reports a case in 1820; Kluge, in 1825, reduced a case of this kind by manipulation with extension; Wathman, in 1826, gives instructions for reducing this dislocation by manipulation; Rust recommends this in 1826; Colombat gives similar advice in 1830; Collins suggests this mode in 1833; Desprez in 1835; Fischer, Mahr and Clarke, in 1840. In 1851, Dr. W. W. Reid, of Rochester, New York, published a report of cases, the first in 1844, thus treated by himself. Many practitioners in more recent times have reported cases of reduction by manipulation. The profession are indebted to Dr. W. W. Reid for his clear elucidation of the principle upon which manipulation is founded, proving by collateral evidence that the chief impediments to reduction were not owing to muscular contraction, but *indirect* action of the muscles that are in a state of tension by the abnormal position of the bone. He has thus thrown a clearer light upon an hitherto obscure point, and placed manipulation upon the high vantage ground of action, justified by a careful and rigid study of the elements observed in this lesion. The question may here be pertinently asked, why has not manipulation received that general adoption which its advocates claim it so eminently merits? The reflections of Hamilton upon this point appear to the writer so excellent as to be readily admitted as highly plausible, if not received as a just exposition. "That some conclusion ought to be drawn from the circumstances, that since the time of Hippocrates to the present day, manipulation has been occasionally recommended, and successful examples reported. The reduction being accomplished in most instances by processes identical, or nearly so with those now adopted, yet generally the writers appear to have been ignorant of what has been done before, and, indeed, they have generally avowed their belief that the method suggested by themselves was new and original. Possibly the slowness to establish, and total inability to sustain and perpetuate a reputation, was not the fault of the method, and had nothing to do with its failures." Extension is a time-honored mode of reduction, and claims for its support names sufficient to create a dynasty of opinion: Sir A. Cooper, S. Cooper, Boyer, Vidal, B. Cooper, Fergusson, Miller, Pirrie, Erichsen, Petit, Duverney, Dorsey, Gibson, Mott and Van Buren.

Hippocrates has expressed his preference of mode in the following words, after recommending the suspension of the man by the injured limb from a gallows, describes it as "a good, proper and natural mode of reduction, and one which has something of display in it if any one takes delight in such ostentatious modes of procedure."

*The Relative Advantages of Manipulation and Extension.*—In manipulation there is less compression of the muscles.

The leverage power of the femur is its greatest advantage, if properly and judiciously applied.

The means are always available, and does not tire and exhaust the muscles, but places the bone in a position when the normal action of the muscles will easily restore the bone to its proper position. Inflammation, suppuration and caries are more likely to follow the reduction by extension, because the amount of violence produced is, *pari passu*, with the greater leverage, obtained by applying the force to the leg, through the mechanical advantages of pulleys. In manipulation the head of the bone is liable to assume different positions, and thereby rupture the various soft tissues. The avowed purpose of extension is to exhaust muscular contractility, and in so doing, are we not more likely to do violence to the muscular, vascular and nervous tissues of the joint? Any one who has witnessed the application of Dr. Jarvis' apparatus, or the pulleys and its adjuncts, by Mr. L'Estrange, must admit that these means are more potent for injury to surrounding tissues; and if we receive the views of Dr. W. W. Reid, extension *per se* is unnecessary and illogical.

*Snap of Reduction.*—Heard, when the muscles have not been paralyzed by chloroform, or tired by long-sustained tension. The case herein reported was reduced without any snap of muscular contraction. Was it the result of the large amount of chloroform administered, or the result of muscular exhaustion, from the long and fruitless contraction of the triceps adductor? In this case the soldier was very muscular in habit, and we may venture to say, without much fear of contradiction, that the degree of snap is generally *pro rata* with the extent of anæsthesia produced. A very interesting and decisive experiment in this regard was performed by M. Case, of Strasburg. He states that a limb can be exempted from anæsthesia, or its extent controlled during inhalation, by merely compressing the main artery that supplies the limb. (Annuaire de Therap, 1850). Again, the answer may be offered, that under chloroform the uterus maintains its expulsive efforts, and why should the reflex action in the muscles of a limb be annulled for the time? The reflex action of the uterus is held in abeyance, whilst its expulsive efforts are continued under the direction of the ganglionic system of nerves.

Thanks are eminently due Surgeon E. S. Gaillard for many facts presented in this paper.

